# Supplementary material for: The interactome of intact mitochondria by cross-linking mass spectrometry provides evidence for coexisting respiratory supercomplexes
Source: Mol Cell Proteomics. 2017 Dec 8;17(2):216–32. doi: 10.1074/mcp.RA117.000470 (PMC5795388; doi:10.1074/mcp.RA117.000470)
Supplement: Supplemental Data [file supp_RA117.000470_133922_0_supp_23978_fzffwf.zip › spectra_annotation/mito_DR_spectra_annotation/243-1-49-1-15-1.pdf]

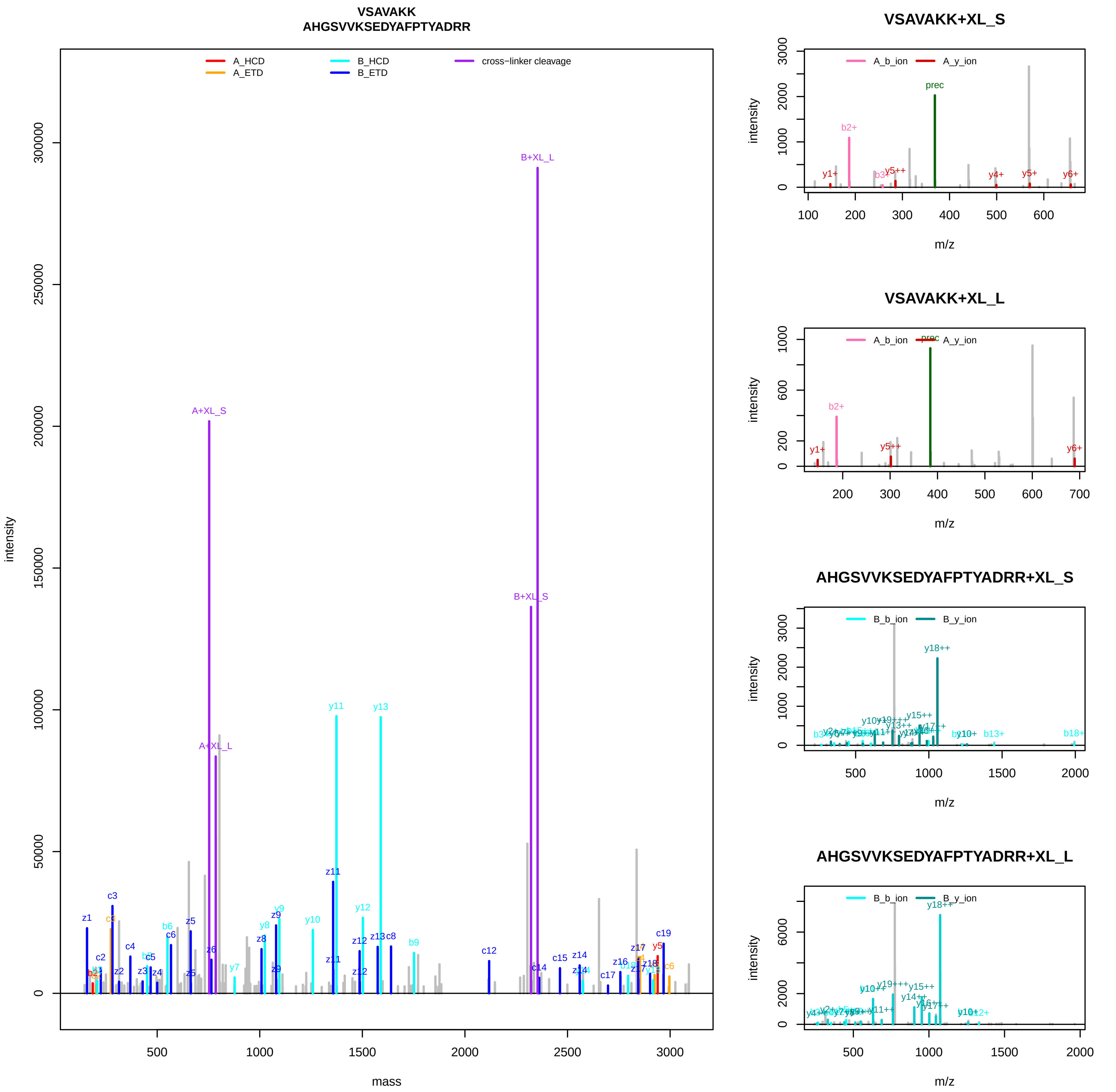
VSAVAKK
AHGSVVKSEDYAFPTYADRR
A_HCD
A_ETD
B_HCD
B_ETD
cross−linker cleavage
intensity
mass
0
50000
100000
150000
200000
250000
300000
500
1000
1500
2000
2500
3000
B+XL_L
A+XL_S
B+XL_S
A+XL_L
y11
y13
z11
y12
y10
y9
z9
y8
z8
z12
z13
c8
b9
c12
c14
c15
z14
z16
z17
c17
c19
y5
z18
c6
c3
z1
c2
c4
z2
z3
z4
c5
b6
c6
z5
z6
y7
VSAVAKK+XL_S
A_b_ion
A_y_ion
prec
b2+
b3+
y1+
y5++
y4+
y5+
y6+
m/z
VSAVAKK+XL_L
y1+
y5++
y6+
AHGSVVKSEDYAFPTYADRR+XL_S
B_b_ion
B_y_ion
y18++
y15++
y13++
y17++
b10+
b13+
b18+
AHGSVVKSEDYAFPTYADRR+XL_L
y10++
y19+++
y15++
y14++
y16++
y17++
y10+
y12+
